# Longitudinal study on MRI and neuropathological findings: Neither DSC-perfusion derived rCBV_max_ nor vessel densities correlate between newly diagnosed and progressive glioblastoma

**DOI:** 10.1371/journal.pone.0274400

**Published:** 2023-02-01

**Authors:** Eike Steidl, Katharina Filipski, Elke Hattingen, Joachim P. Steinbach, Gabriele D. Maurer

**Affiliations:** 1 Institute of Neuroradiology, Goethe University Hospital, Frankfurt am Main, Germany; 2 German Cancer Consortium (DKTK) and German Cancer Research Center (DKFZ), Heidelberg, Germany; 3 Institute of Neurology (Edinger Institute), Goethe University Hospital, Frankfurt am Main, Germany; 4 Frankfurt Cancer Institute (FCI), Frankfurt am Main, Germany; 5 Dr. Senckenberg Institute of Neurooncology, Goethe University Hospital, Frankfurt am Main, Germany; IRCCS Giovanni Paolo II Cancer Hospital, ITALY

## Abstract

**Introduction:**

When evaluating MRIs for glioblastoma progression, previous scans are usually included into the review. Nowadays dynamic susceptibility contrast (DSC)-perfusion is an essential component in MR-diagnostics of gliomas, since the extent of hyperperfusion upon first diagnosis correlates with gene expression and survival. We aimed to investigate if this initial perfusion signature also characterizes the glioblastoma at time of progression. If so, DSC-perfusion data from the initial diagnosis could be of diagnostic benefit in follow-up assessments.

**Methods:**

We retrospectively identified 65 patients with isocitrate dehydrogenase wildtype glioblastoma who had received technically identical DSC-perfusion measurements at initial diagnosis and at time of first progression. We determined maximum relative cerebral blood volume values (rCBV_max_) by standardized re-evaluation of the data including leakage correction. In addition, the corresponding tissue samples from 24 patients were examined histologically for the maximum vessel density within the tumor. Differences (paired t-test/ Wilcoxon matched pairs test) and correlations (Spearman) between the measurements at both timepoints were calculated.

**Results:**

The rCBV_max_ was consistently lower at time of progression compared to rCBV_max_ at time of first diagnosis (p < .001). There was no correlation between the rCBV_max_ values at both timepoints (r = .12). These findings were reflected in the histological examination, with a lower vessel density in progressive glioblastoma (p = .01) and no correlation between the two timepoints (r = -.07).

**Conclusion:**

Our results suggest that the extent of hyperperfusion in glioblastoma at first diagnosis is not a sustaining tumor characteristic. Hence, the rCBV_max_ at initial diagnosis should be disregarded when reviewing MRIs for glioblastoma progression.

## Introduction

The revised Response Assessment in Neuro-oncology (RANO) criteria for the evaluation of treatment response in patients with glioblastoma have been implemented more than a decade ago [1]. To this day, they are the basis for decisions on further diagnostic and therapeutic procedures in and outside of clinical studies [2,3]. In addition to corticosteroid use and the clinical condition of the patient, the RANO criteria rely on morphologic imaging features, in particular the assessment of contrast enhancement and T2/FLAIR hyperintensity in comparison to previous MRI scans. Given the limitations of this morphological approach especially with regard to the differentiation of tumor progression and radionecrosis [4], various additional imaging techniques have been investigated. While metabolic imaging with MR-spectroscopy and O-(2-[^18^F]fluoroethyl-)-L-tyrosine PET can provide significant further information [5–8], the most widely available and commonly used modality is the dynamic susceptibility contrast (DSC) MR-perfusion technique [9]. In glioblastoma, the most frequently investigated DSC-perfusion parameter is the cerebral blood volume, normalized to the contralateral white matter (rCBV) [10]. The rCBV is a surrogate parameter for the microvasculature of the tissue and has been shown to correlate with the microvascular density, the microvascular area and the vessel morphology derived from the histological examination of image-guided biopsy samples [11–13]. Values are increased in glioblastomas as they overexpress proangiogenic cytokines like vascular endothelial growth factor (VEGF) and display enlarged, irregular and leaky vessels [14]. Nonetheless, the extent of the rCBV increase is very variable [10], yet associated with the expression of genes such as the epidermal growth factor receptor (*EGFR*) and mutant isocitrate dehydrogenase (*IDH*) [15–18]. Several studies also indicated that rCBV at initial diagnosis is predictive of poor patient survival independent from other known prognosticators [15–18]. The measurement and interpretation of rCBV values at a single timepoint is the common approach, but there have also been more recent efforts to improve the diagnostic accuracy for detecting glioblastoma progression by analyzing longitudinal data [19–21]. Besides, repeated DSC-perfusion measurements have been employed to monitor effects of radiochemotherapy and anti-angiogenic agents [22–25]. Despite these efforts, it is currently unclear whether the extent of hypervascularization is a permanent feature of an individual tumor, as the association with tumor molecular characteristics and patient survival might suggest. In particular, there is little data on rCBV in glioblastoma at the time of tumor progression versus initial diagnosis, with two studies reporting lower rCBV values overall at progression [26,27]. To our knowledge, it has not been investigated whether and how rCBV values at first diagnosis and progression correlate. Consequently, it is not known whether high initial rCBV values identify glioblastomas with a stronger and persisting tendency towards neoangiogenesis. However, this information is not only of scientific interest, but also relevant in everyday clinical practice when the initial imaging is used to assess subsequent examinations.

Therefore, the aim of this study was to investigate whether DSC-perfusion derived rCBV values correlate between newly diagnosed glioblastomas and the same tumors at first progression. We sought to minimize potential confounders by analyzing technically uniform data in a homogeneous patient cohort, as well as by adding histologic analysis for correlative analysis.

## Methods

### Study design and patient cohort

This retrospective analysis was approved by the scientific board of the University Cancer Center Frankfurt and the local ethics committee (UCT-22-2020). All patients were cared for in our neuro-oncology department We screened our institutional database for patients meeting the following three criteria:

1. Neuropathologically confirmed diagnosis of an *IDH* wildtype glioblastoma
2. At least 18 years old at initial diagnosis
3. DSC-perfusion measurements using identical protocols and acquired at the same 3 Tesla MRI scanner at both initial diagnosis and first time of progression. As a result of this criterion, recruitment was set to begin in January 2011, after a new 3 Tesla MRI scanner had been installed.

When available, samples from patients who met these criteria and who had been treated surgically for their first glioblastoma progression were reevaluated neuropathologically. Both the primary and the progressive tumors were examined.

### Imaging protocol and DSC-perfusion analysis

All examinations were conducted on 3 Tesla MRI scanners (Verio^®^ or Skyra^®^; Siemens, Erlangen, Germany). The anatomical MRI included T2-weighted sequences and T1-weighted sequences before and after administration of contrast agent. The acquisition parameters for the DSC-perfusion measurements were equal for both scanners (gradient-echo echoplanar imaging; time-to-echo, 30 ms; time-to-repeat, 1790 ms; flip-angle, 90°; slice thickness, 3 mm; 50 dynamic scans, field of view, 230 x 230 mm; matrix, 128 x 128). Measurements were performed either with or without application of a contrast agent pre-bolus before administering the intravenous main bolus (gadolinium-based agent, 0.1 mmol/kg bodyweight; infusion rate, 4 ml/s followed by 21 ml of 0.9% w/v sodium chloride). As specified above, the acquisition schemes had to be identical in individual patients for measurements at initial diagnosis and first progression. Measurements with insufficient contrast agent bolus according to the time-signal curves or severe motion artifacts were excluded.

**Fig 1:**
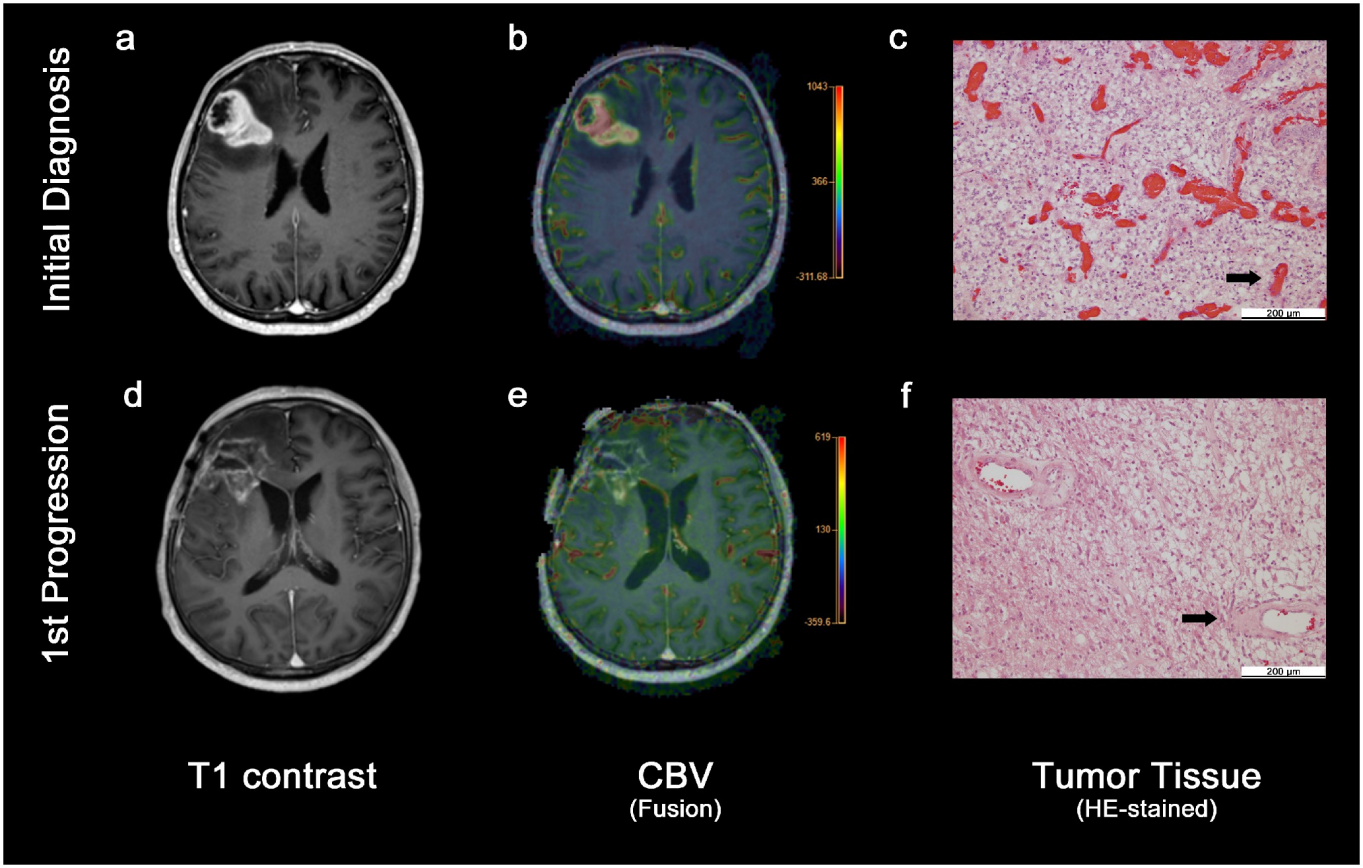
Patient example. MR-images and tumor tissue of an exemplary patient at initial diagnosis (first row) and first tumor progression (second row). T1-weighted contrast-enhanced images (a and d), cerebral blood volume (CBV) maps as transparent overlays on the T1-weighted contrast-enhanced images (b and e) and hematoxylin and eosin (HE) stained tumor tissue (c and f) are shown. Color bars in b and e display the CBV range. Arrows in c and f indicate exemplary vessels within the tumor. Both the CBV and the vessel density in the tumor were notably higher at the initial diagnosis in comparison to the first progression.

The raw data of all perfusion measurements was homogeneously reanalyzed using the MR Neuro Perfusion application of the Philips IntelliSpace^®^ software toolbox. The automated software uses the Boxerman–Weisskoff approach to assess brain perfusion curves that have been corrected for leakage of contrast agent into the tissue [28]. The calculated perfusion maps were used as a layover on contrast enhanced T1 and the T2 datasets. This allowed for the exclusion of vessels and the identification of the tumor margin. The region of maximum CBV within the tumor was then visually assessed and mapped in consensus by experienced radiologists E.H. and E.S.. A correlating region of interest (ROI) of identical size in the contralateral, normal appearing white matter was used for normalization and calculation of the leakage corrected maximum CBV (rCBV_max_). An exemplary case is shown in Fig 1. To minimize biases, first, all measurements from initial diagnoses were analyzed consecutively, then all measurements from the first time of progression.

### Histologic analysis

An experienced neuropathologist (K.F.), who was blinded to the results of the MR-perfusion measurements, analyzed all available tissue samples, again consecutively rating all samples from initial diagnoses before rating the samples from first time of progression. Formalin-fixed and paraffin-embedded tumor tissue sections were stained with hematoxylin and eosin (HE) and assessed visually. The maximum vessel density (vessels/mm^2^) was calculated from the average number of vessels in three fields of view (FOV) at 20-fold (for resections; 1 FOV = 0.95 mm^2^) or 40-fold magnification (for biopsies; 1 FOV = 0.196 mm^2^) in the tumor area displaying the highest density of vessels. In addition, the dominating vessel size (small; medium; large) and the dominating vessel wall characteristic (sclerosed/hyalinized; microvascular proliferate-like; slender/non-microvascular proliferate-like) were recorded. Fig 1 depicts an exemplary case.

### Statistics

Statistical testing was done employing either SPSS^®^ Statistics 28 (IBM, Armonk, NY, USA) or a commercially available inhouse software called BiAS^®^ (https://www.bias-online.de/). The Kolmogorov-Smirnov-Lilliefors test was used to test for normal distributions; the values for CBV_max_ and patient age followed normal distribution while all other variables did not. Differences in rCBV_max_ values and vessel densities between primary and progressive tumors were assessed either by the paired t-test or the Wilcoxon matched pairs test. Differences in the time to progression between subgroups were analyzed by Wilcoxon-Mann-Whitney-test. Correlations between the values measured in newly diagnosed and progressive glioblastomas were calculated using the Spearman’s rank correlation. Even for the rCBV_max_ values, that followed normal distribution, the assumption of a linear correlation as a prerequisite for the Pearson correlation did not seem to be appropriate and the Spearman’s rank correlation was used instead. A p-value of less than 0.05 was deemed to indicate significance.

## Results

### Patients

We identified 65 patients who fulfilled all inclusion criteria and whose DSC-perfusion measurements qualitatively met our requirements (see also the flow diagram in S1 Fig). Following resection or biopsy between July 2010 and March 2020, 60 patients (92%) had received tumor-specific treatment, i.e. always radiotherapy and mostly (n = 56, 86%) antineoplastic drugs. For varying reasons, including early progression, trauma requiring surgery and the use of alternative medicine, 5 patients (8%) did not have adjuvant therapy. Detailed data on the patient characteristics as well as first-line treatments are given in Table 1.

**Table 1:** Patient and tumor characteristics (n = 65).

		%
Age at initial diagnosis [years; mean ± standard deviation]	57.8 ± 10.5	
Gender [number; male/female]	48/17	74/26
Type of surgery at initial diagnosis [number; resection/biopsy]	51/14	78/22
*MGMT*^a^ promoter methylation status [number; unmethylated/methylated/n.a.^b^]	41/20/4	63/31/6
Tumor location by side [number; right/left/bilateral]	36/28/1	55/43/2
Brain regions affected, initial MRI^c^ [number]		
Temporal/frontal/parietal/occipital lobe	27/16/15/12	36/22/20/16
Insula/thalamus/basal ganglia	1/2/1	1/3/1
Tumor-specific treatment [number; yes/no]	60/5	92/8
Radiotherapy [number; yes/no]	60/5	92/8
Drug-based tumor therapy [number; yes/no]	56/9	86/14
Alkylating chemotherapy	51	78
Temozolomide/temozolomide and lomustine	51/5	78/8
Palbociclib/other^d^	3/3	5/5
Tumor treating fields	5	8
Time to progression^e^ [days; median, range]	227, 43−2227	
Time between end of first-line treatment and progression [days; median, range]	26, 0−1837	
Confirmation of tumor progression [number; surgery^f^/imaging]	31/34	48/52
Overall survival^g^ [days; median, range]	513, 70−2677	

^a^ *MGMT*, O6-methylguanine-DNA methyltransferase.

^b^ A glioblastoma may extend over more than one region, with the major localization(s) listed here.

^c^ n.a., not available.

^d^ other, comprising one patient each treated with temsirolimus, marizomib, and bevacizumab/irinotecan, respectively, in the context of clinical trials.

^e^ Time to progression, defined as the time from initial surgery to first tumor progression according to RANO criteria (date of either surgery or, if none was performed, the imaging).

^f^ Surgery included 30 resections and one biopsy.

^g^ Overall survival, defined as the time from initial surgery to death; all 65 patients were deceased at the time of the analysis.

Fifty-two patients received the standard 6-week radiotherapy with a total dose of 60 Gy [29] In two patients, radiotherapy planned up to 60 Gy was terminated prematurely at a total dose achieved of 44 Gy and 50 Gy, respectively. Six patients received hypofractionated irradiation with a total dose of 40 Gy [30,31]. All of these patients were older (mean 72.3 years, standard deviation 7.1) and had a tumor lacking O6-methylguanine-DNA methyltransferase (*MGMT*) promoter methylation. The diagnosis had been made after resection of the tumor in two cases and after biopsy in four cases. Chemotherapy (temozolomide) was administered in the first-line treatment of only two of the six patients; the other four were treated with radiation alone. The median overall survival of these six patients was 260 days (range 133–860).

### DSC-perfusion

**Fig 2:**
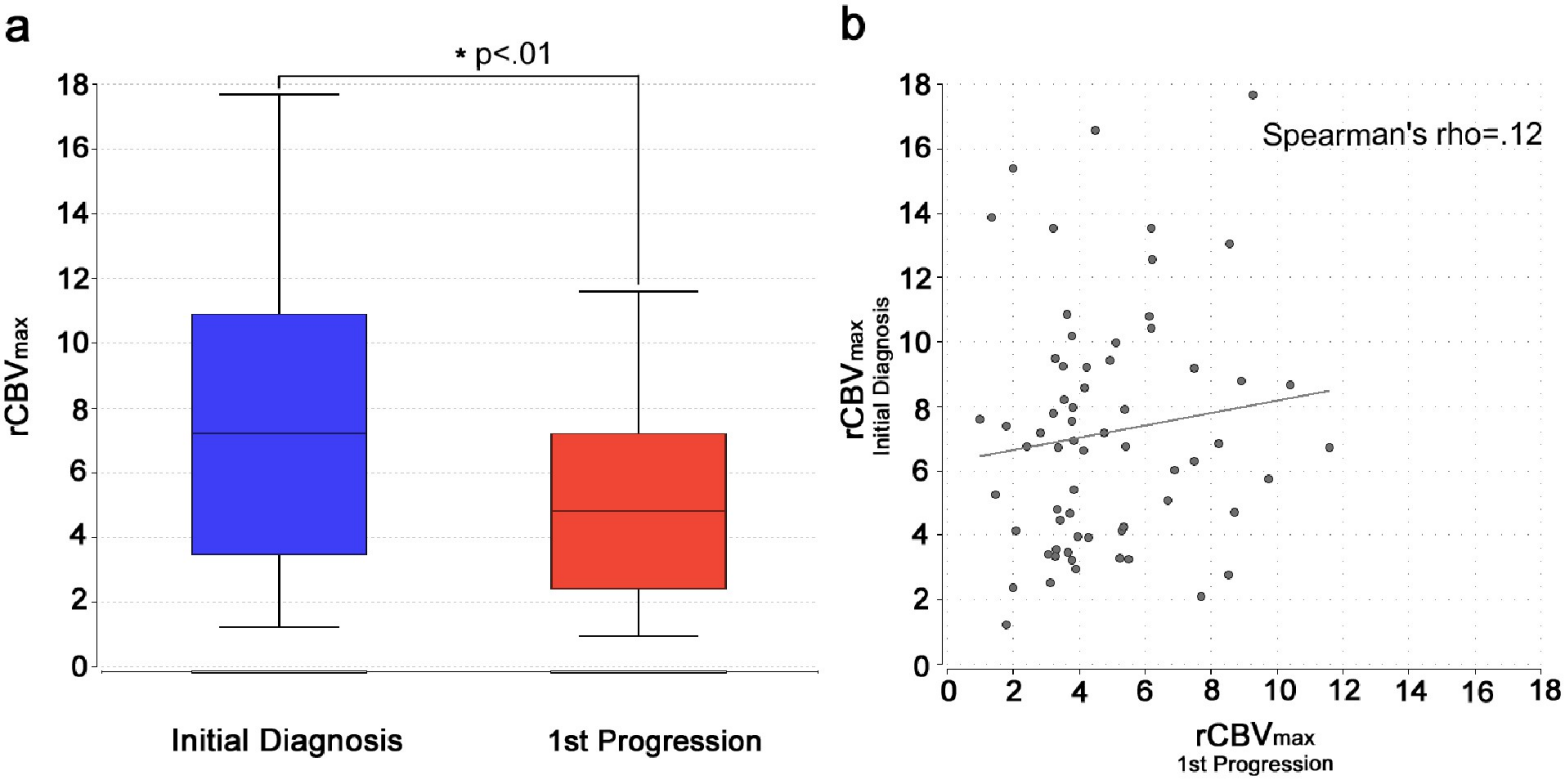
rCBV_max_ at initial diagnosis and progression. Relative cerebral maximum blood volume in the tumor (rCBV_max_) significantly (p < .01) decreasing from initial diagnosis to first progression displayed as box-plots (whiskers indicate the standard deviation, the horizontal line within the boxes indicates the mean) (a) and missing correlation (Spearman’s rho = .12) of the rCBV_max_ values at initial diagnosis and first progression displayed as scatter plot with regression line (b).

The rCBV_max_ values measured in glioblastoma at initial diagnosis were significantly higher than the corresponding values in progressive tumors (mean 7.2 vs. 4.9; p < .001, paired t-test; Fig 2A). Noteworthy, this effect was mainly seen in patients who had undergone resection (n = 51; mean 7.6 vs. 4.6; p < .001, paired t-test) and not biopsy only (n = 14; mean 5.8 vs. 5.6; p = .83, paired t-test; S2A and S2B Fig). Patients who underwent biopsy only had a significantly shorter time to progression (median 5.7 vs. 7.9 month; p = .02, Wilcoxon-Man-Whitney-test). Within the subgroups the change in rCBV_max_ did not correlate significantly with the time to progression (biopsy only subgroup: Spearman’s rho = -.004, p = .99; resection subgroup: Spearman’s rho = .26, p = .06). Beyond the difference in rCBV_max_ magnitudes, there was no significant correlation between the rCBV_max_ values at initial diagnosis and progression (Spearman’s rho = .12, p = .33; Fig 2B). The same held true when separately analyzing the subgroups of patients who had undergone either a resection or biopsy only at initial diagnosis (Spearman’s rho = .12/.22; p = .40/.44; S2C and S2D Fig). There was no significant difference between the ROI sizes at initial diagnosis and first progression (median ROI size 34 vs. 30 mm^2^, p = .41, Wilcoxon matched pairs test).

### Histology

**Fig 3:**
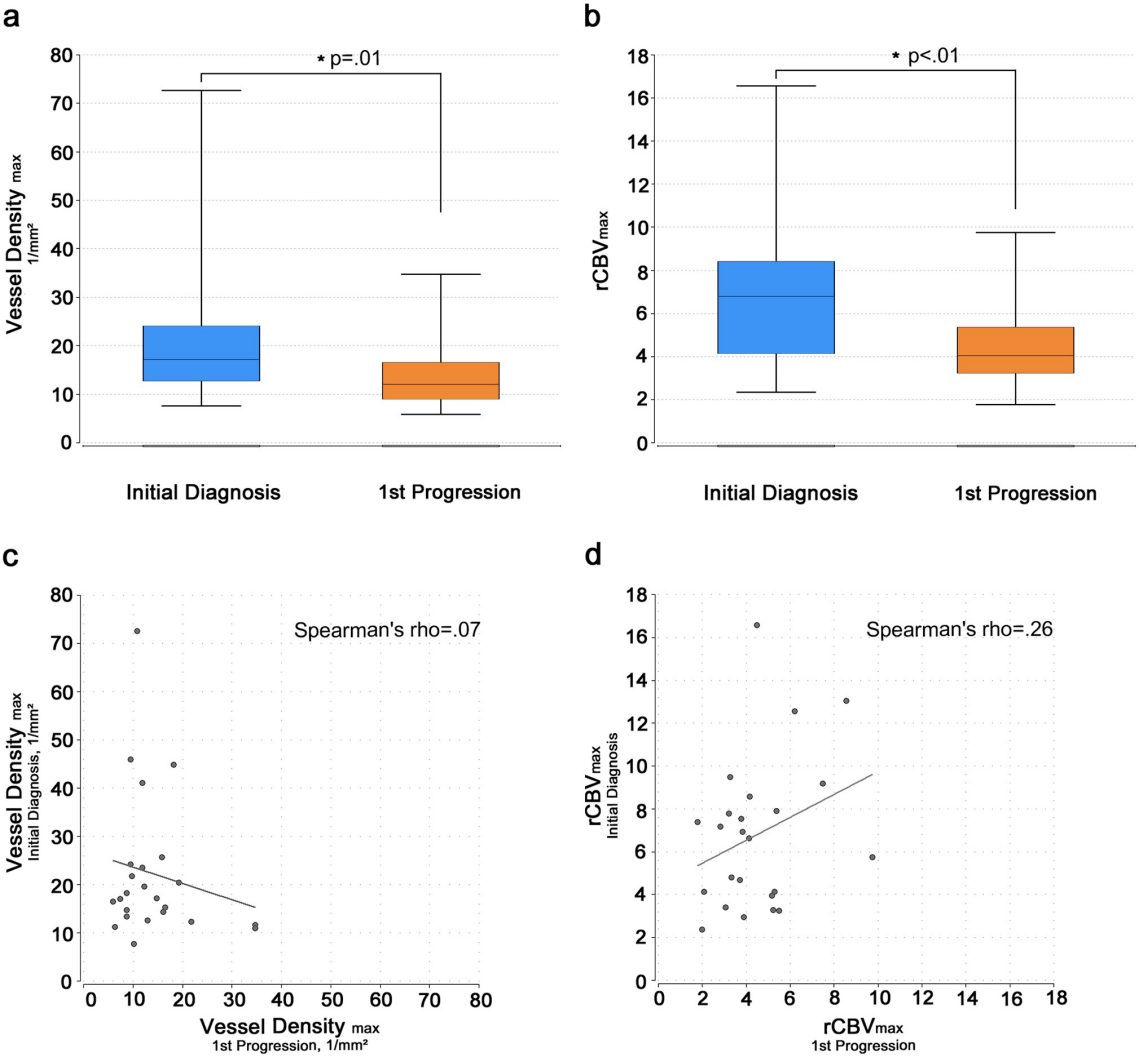
Vessel densities and corresponding rCBV_max_ in patients with available tumor tissue (n = 24). Maximum vessel densities in the tumor (1/mm^2^, a) and corresponding relative cerebral maximum blood volume in the tumor (rCBV_max_, b) significantly (p = .01/p < .01) decreasing from initial diagnosis to first progression displayed as box-plots (whiskers indicate the first and third quartile for a and the standard deviation for b, the horizontal line within the boxes indicates the median for a and the mean for b) for the subgroup of patients with available tumor tissue at both timepoints (n = 24). Missing correlations (Spearman’s rho = .07/.26) of the maximum vessel densities in the tumor (1/mm^2^, c) and the corresponding rCBV_max_ values (d) at initial diagnosis and first progression depicted as scatter plot with regression line.

Tumor tissue samples from both the initial diagnosis (22 resections/ 2 biopsies) and the first progression (all resections) were available for 24 patients. Twenty-two of these patients had undergone adjuvant radiochemotherapy in between surgeries, two patients had not received adjuvant therapy. As for the rCBV_max_ values, the maximum vessel densities were significantly lower in progressive glioblastomas than in the corresponding newly diagnosed tumors (median 11.9 vs. 17.1 vessels/mm^2^; p = .01, Wilcoxon matched pairs test; Fig 3A; mean rCBV_max_ for these 24 patients 4.5 vs. 6.8; p < .01, paired t-test; Fig 3B). Again, similar to the results of the DSC-perfusion analysis, the vessel densities of initial diagnosis and first progression did not correlate (Spearman’s rho = -.07, p = .73; Fig 3C; Spearman’s rho for the rCBV_max_ values in these 24 patients = .26, p = .22; Fig 3D), and the change in vessel density did not correlate with time to progression (Spearman’s rho = -.11, p = .61). Remarkably, the vessel densities in the glioblastomas of the two patients who had not received adjuvant treatment also changed from the first to the second tumor resection (11.2 to 34.4 vessels/mm^2^ and 46.0 to 9.5 vessels/mm^2^, respectively). The dominant vessel size switched in 15/24 cases from initial diagnosis to first progression, mostly from small to medium. The dominant vessel wall characteristic changed in 8/24 patients, with none of the samples displaying a sclerosed/hyalinized vessel wall type either before or after first-line therapy.

## Discussion

Our study addressed the question whether the extent of the DSC-perfusion derived rCBV_max_ increase in glioblastoma at initial diagnosis is a permanent tumor characteristic that can be retrieved when the tumor is regrowing or progressing.

In accordance with the two studies reporting rCBV values in glioblastoma both at initial diagnosis and first progression [26,27], we found a significant decrease in the rCBV_max_ from initial diagnosis to first progression. This finding was reflected by a simultaneous decrease in the histologic vessel density. Our subgroup analysis revealed that the reduction was mostly driven by patients who had undergone resection upon initial diagnosis and was not detectable in patients who had received a biopsy only (S2A and S2B Fig). The rate of adjuvant treatment with radio- and/or alkylating chemotherapy was comparable in both subgroups but patients in the biopsy only subgroup had a significantly shorter time to progression. When analyzed separately in both subgroups, the change in rCBV_max_ did not show a significant correlation to the time to progression. Besides the possibility of more aggressive tumors cumulating in the biopsy only subgroup, one can assume that the surgical cytoreduction had an impact on the rCBV_max_ values. This is noteworthy given the paucity of data linking the size of a glioblastoma to its perfusion properties, with one small study (n = 14) actually reporting an inverse correlation of tumor size and CBV [32].

As mentioned in the introduction section, several studies have connected the initial rCBV in glioblastoma to gene expression patterns. Heiland et al. correlated higher rCBV values with enrichments in the EGF pathway and a mesenchymal signature, that in turn are regarded as characteristics of a more aggressive glioblastoma phenotype [33,34]. In view of the reported association with overall survival [15–18], it is reasonable to assume that tumors with a high initial rCBV_max_ also tend to have a marked hyperperfusion when they regrow. Yet, we did not find any correlation between the rCBV_max_ measurements from initial diagnosis and first progression (Fig 2B). As correlations on an individual level might be diluted by several technical factors, we considered tangible confounders in our study design, restricted the analysis to a homogeneous cohort with technically identical measurements at both timepoints and completely re-analyzed the raw data. Also, a significant difference in the sizes of the manually drawn ROIs between the two timepoints could be excluded. Our finding was substantiated by our histologic examination of vessel densities in the tumors, which also showed no correlation between the samples from the initial diagnosis and the first progression. Here, a change of the dominating vessel size was observed in 63% of the samples. The missing correlation of the rCBV_max_ values was independent of the initial surgical approach (biopsy or resection, Fig 2), meaning even though resections seemed to lead to reduced rCBV_max_ values in many tumors, they were likely not the primary reason for the missing correlation. An obvious explanation could be the effect of irradiation and chemotherapy. Although we did not see an increase in sclerosed/hyalinized vessel walls as an indicator of radiation damage in our cohort, the impact of both radio- and chemotherapy on glioblastoma mutational load, genetic mutations and epigenetic modifications is becoming increasingly understood [35,36]. In particular, Scholz et al. [37] previously also demonstrated a decrease in glioblastoma vessel density after either radiochemotherapy or radiochemotherapy plus bevacizumab and described concurrent changes in the angiopoietin/Tie2 signaling pathway. Furthermore, to our knowledge, it is not known to what extent rCBV values in untreated glioblastoma remain stable over time. As glioblastomas are aggressive tumors with average growth rates of 1.4%/day [38] and regular development of necrosis, rCBV_max_ values might fluctuate despite an underlying tendency to a more pronounced hypervascularization in certain tumors. Since this was not the main question of our study, it can only be noted that changes in rCBV_max_ and vessel density were also found in the two patients who had not received any adjuvant treatment in between the two tumor resections. As a conclusion, the extent of the rCBV_max_ increase in glioblastoma cannot be considered a persisting tumor feature. Hence, especially in individual cases, the initial perfusion properties of a tumor should be disregarded when reviewing follow-up MRI scans. As the diagnostic value of MR-perfusion might even increase with growing standardization [39], cut-off based approaches remain key for the interpretation of DSC-perfusion when tumor progression is suspected [10,40].

### Limitations

A major limitation of our study is its retrospective nature, and many confounders and biases could be considered. Both the initial population and the cohort ultimately analyzed do not represent the entirety of all glioblastoma patients. In the first step, we searched for patients with a first progression according to the RANO criteria. Thus, the data of patients who were treated exclusively with supportive care after diagnosis, i.e. in particular individuals in a poorer general condition and/or with a more aggressive disease, or who did not present to us for follow-up, were not captured. Not all patients receive follow-up imaging at our center, but many have their MRI performed close to home prior to presentation. Given the noncomparable technical requirements, these patients could not be included in the analysis. Also, MR-perfusion is not routinely performed during follow-up of brain tumors in all outpatient radiology services [41,42]. Therefore, after exclusion of numerous patients in whom either no or insufficient perfusion data were available, only 65 cases ultimately remained for our study (S1 Fig). While this main cohort was mostly defined by tumor type and technical requirements, the availability of tumor tissue was linked to the clinical decision to perform surgery. However, the recommendation of surgery is based, among other factors, on the patient’s general condition, symptoms, and tumor location. Thus, the histologic data represent only a subset of glioblastomas and cannot be readily extrapolated to our cohort of 65 patients. We did not perform extensive subgroup analyses, for example, by irradiation dose, because the respective subgroups are small and subject to inherent biases. For example, hypofractionated irradiation, as in 6 of our 65 patients, is more likely to be applied to older patients in poorer general health, and the absence of *MGMT* promoter methylation is associated with comparatively shorter survival. Extensive work and recommendations exist on tumor and patient characteristics and their relevance to prognosis and therapy [2,43], and such issues were not the focus of our imaging-based study. Due to the retrospective approach, we were not able to match the locations of the histologic and MR-perfusion “hot spots”, which would have allowed for a more detailed analysis. Also, the employed perfusion protocol is not fully in line with newer consensus recommendations [44], yet the impact on the actual stability of the measurements is likely limited. Lastly, we did not exclude patients who had received additional therapies like tumor-treating fields whose impact on tumor perfusion is not well investigated. By means of the perfusion measurement examined here, the macrovasculature can be judged better than the microvascular network [45]. Indeed, in glioblastoma progression and as a result of the selection and adaptation processes, the latter seems to be altered earlier and more profoundly than the former [46]. However, the DSC-perfusion measurement is frequently part of the routine protocol in the MRI of glioblastoma and the present study was initiated with the question of whether the longitudinal evaluation of rCBV_max_ could provide a diagnostic benefit in everyday clinical practice.

## Supporting information

S1 Fig Flow chart depicting the patient selection process. The relatively large number of patients for whom MR-perfusion data were not available at at least one timepoint is mainly due to the fact that many patients had their follow-up MRI examinations performed at external facilities closer to their homes.
(TIF)

S2 Fig Individual data. Relative cerebral maximum blood volume in the tumor (rCBV_max_) significantly (p < .001) decreasing from initial diagnosis to first progression in patients with initial resection but not in patients with biopsy only. Ladder-plots with individual rCBV_max_ values for every patient in the two subgroups and a red line indicating the mean (a). Missing correlations (Spearman’s rho = .12/.22) of the rCBV_max_ values at initial diagnosis and first progression in both subgroups displayed as scatter plots with regression lines (b and c).
(TIF)
