# Supplementary figures and images for: Longitudinal study on MRI and neuropathological findings: Neither DSC-perfusion derived rCBVmax nor vessel densities correlate between newly diagnosed and progressive glioblastoma
Source: PLoS One. 2023 Feb 1;18(2):e0274400. doi: 10.1371/journal.pone.0274400 (PMC9891512; doi:10.1371/journal.pone.0274400)

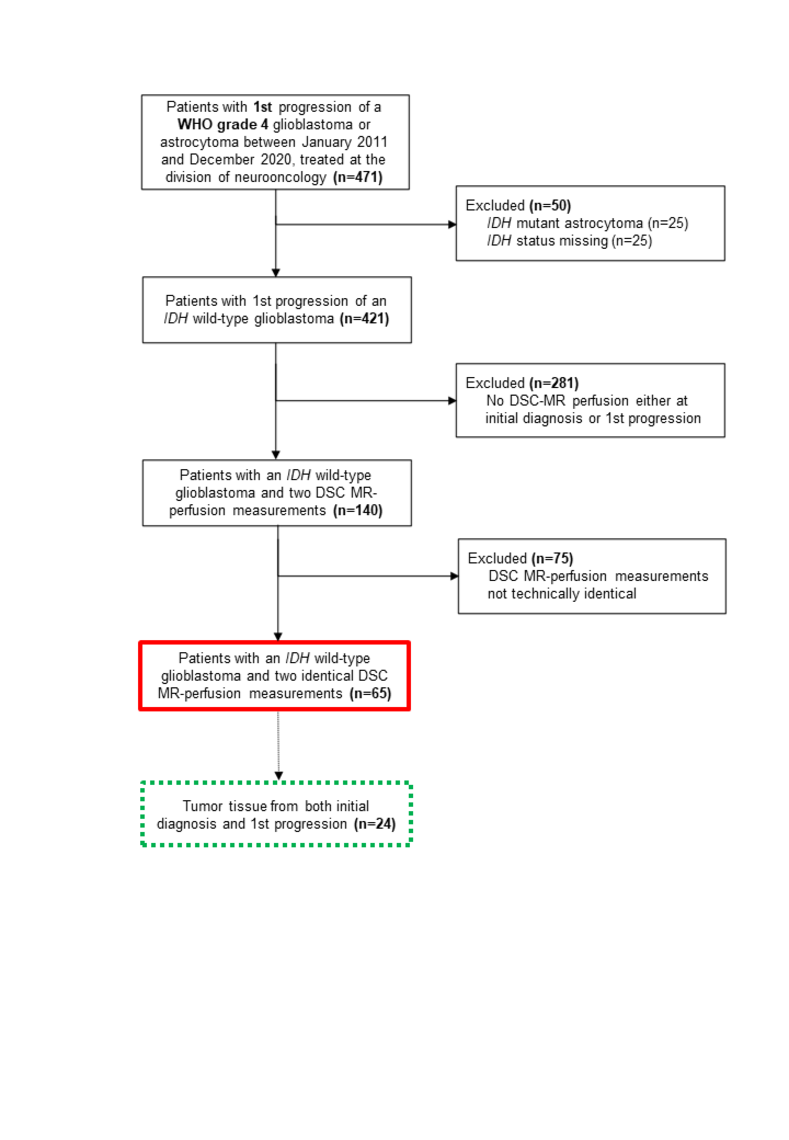

Supplement: S1 Fig — The relatively large number of patients for whom MR-perfusion data were not available at at least one timepoint is mainly due to the fact that many patients had their follow-up MRI examinations performed at external facilities closer to their homes. (TIF) [file pone.0274400.s001.tif]

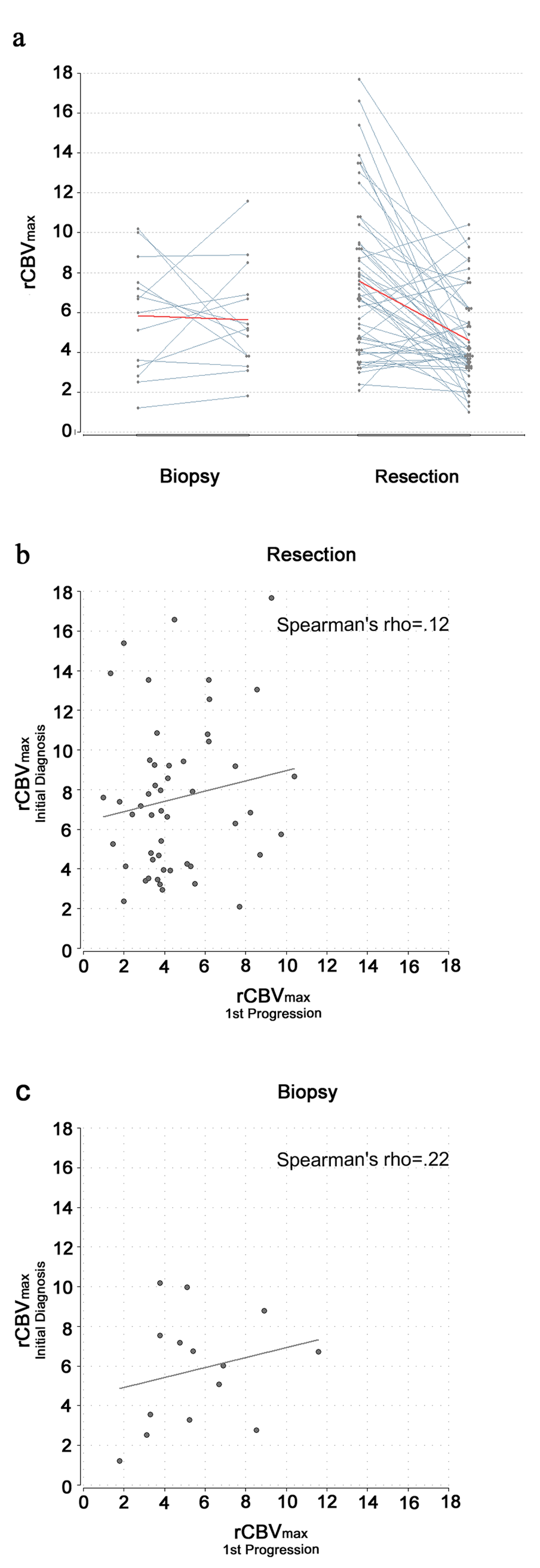

Supplement: S2 Fig — Relative cerebral maximum blood volume in the tumor (rCBVmax) significantly (p < .001) decreasing from initial diagnosis to first progression in patients with initial resection but not in patients with biopsy only. Ladder-plots with individual rCBVmax values for every patient in the two subgroups and a red line indicating the mean (a). Missing correlations (Spearman’s rho = .12/.22) of the rCBVmax values at initial diagnosis and first progression in both subgroups displayed as scatter plots with regression lines (b and c). (TIF) [file pone.0274400.s002.tif]
